# New Directions in the Study and Treatment of Metastatic Cancer

**DOI:** 10.3389/fonc.2018.00258

**Published:** 2018-07-10

**Authors:** Byunghee Yoo, Bryan C. Fuchs, Zdravka Medarova

**Affiliations:** ^1^MGH/MIT/HMS Athinoula A. Martinos Center for Biomedical Imaging, Massachusetts General Hospital, Harvard Medical School, Boston, MA, United States; ^2^Division of Surgical Oncology, Massachusetts General Hospital Cancer Center and Harvard Medical School, Boston, MA, United States

**Keywords:** metastasis, cancer stabilization, cancer microenvironment, cancer therapy, targeted therapy

## Abstract

Traditional cancer therapy has relied on a strictly cytotoxic approach that views non-metastatic and metastatic tumor cells as identical in terms of molecular biology and sensitivity to therapeutic intervention. Mounting evidence suggests that, in fact, non-metastatic and metastatic tumor cells differ in key characteristics that could explain the capacity of the metastatic cells to not only escape the primary organ but also to survive while in the circulation and to colonize a distant organ. Here, we lay out a framework for a new multi-pronged therapeutic approach. This approach involves modifying the local microenvironment of the primary tumor to inhibit the formation and release of metastatic cells; normalizing the microenvironment of the metastatic organ to limit the capacity of metastatic tumor cells to invade and colonize the organ; remediating the immune response to tumor neoantigens; and targeting metastatic tumor cells on a systemic level by restoring critical and unique aspects of the cell’s phenotype, such as anchorage dependence. Given the limited progress against metastatic cancer using traditional therapeutic strategies, the outlined paradigm could provide a more rational alternative to patients with metastatic cancer.

## Metastatic Cancer as a Unique Therapeutic Challenge

The traditional view of tumors has been as homogeneous masses of cells that proliferate uncontrollably and are resistant to pro-apoptotic stimuli. Now, we know that this view is overly simplistic. In fact, tumors are highly heterogeneous assemblies of cells that participate in constant interplay between themselves and a dynamic local microenvironment, continually changing and adapting in the process. The most insidious of these cells are those that acquire the capability to break out of the primary tumor mass, travel through the circulation, and colonize a new vital organ in the process of metastasis. Importantly, these cells are genetically and phenotypically distinct from the majority of the cells in the tumor mass, spawning metastatic lesions that have diverged significantly in their gene expression profile from their respective primary tumors.

Based on this knowledge, it becomes clear that successful cancer therapy has to include a component that specifically targets the metastatic niche. The metastatic niche presents a distinct challenge in terms of molecular biology, local tissue microenvironment, immunological profile, and physicochemical tissue properties that are keys to the success of therapy. This is particularly well established with regard to breast cancer. Numerous studies of the receptor status of patient-matched primary and metastatic tumors have reported high discordance rates. In addition, while gene expression profiling has suggested mostly concordance between primary tumors and lymph node/distant metastases, a small number of genes that differ have also been identified, with a large number of the differences being at the non-coding RNA and epigenetic level. Whole-genome sequencing comparisons have also been performed in individual patients and have found heterogeneity in primary tumors and significant differences in the corresponding metastases. A very comprehensive meta-analysis of this evidence can be found in Kroigard et al. (1). Overall, the majority of the studies reveal therapeutically meaningful discordances between primary tumors and metastases, underscoring the need for analysis of metastatic tissue as a guide to therapy.

The traditional focus on the primary tumor for the development of cancer therapies is partly behind the poor outcomes in cancer patients diagnosed with metastatic disease. Conventional therapies targeted toward the primary tumor cell oftentimes do not affect the metastatic cell and, in fact, may promote metastasis. Such is the case for paclitaxel, cisplatin, anti-androgens, everolimus, and sunitinib. This fact is behind the poor outcomes in patients diagnosed with metastatic disease despite the good prognosis of patients with localized cancer of the same organ of origin (2). It is not surprising that 9 out of 10 deaths from cancer are, in fact, due to metastasis.

## Cancer “Stabilization” Therapy as an Alternative Therapeutic Paradigm

Toward the goal of developing metastases-specific therapies, an important question that needs to be addressed is, what makes a cell capable of leaving the organ of its origin, surviving in the circulation and in its non-native tissue, and even colonizing this tissue, which has immunological, physicochemical, and molecular, genetic, and epigenetic properties distinct from the native organ in which the cell originated. In this sense, metastasis can be seen as a disease of tissue-to-tissue histocompatibility. The hypothesis that we propose is that cells endowed with that capability evolve in response to an adaptive process driven by a cellular “survival instinct.” Specifically, as tumors proliferate uncontrollably, within them arise pockets characterized by inadequate resource supply, due to failure of the tumor vasculature and the tumor stroma/extracellular matrix (ECM) to keep up with the rapidly increasing tumor cell burden. This generates local areas of low pH, high inflammation, and insufficient stromal supportive network necessary to maintain the survival of the tumor cells. As a result, the majority of the tumor cells within these pockets die but few evolve by activating mechanisms that allow them to survive in the absence of abundant nutrient supply, evade immune recognition, and persist without the strong attachment to the ECM. These newly emergent “super-cells” become “refugees” from the primary tumor, invisible to most diagnostic/imaging modalities and resistant to most currently available therapeutic modalities.

Against this conceptual framework, it appears that most traditional therapeutic approaches against cancer are in some ways counterintuitive. Chemotherapy, radiation therapy, antiangiogenic therapy, etc., indiscriminately induce tumor cell apoptosis. This promotes the creation of inhospitable pockets within the primary tumor and, consequently, stimulates the evolution of aggressive, metastatic “super-clones.” To our knowledge, this aspect of tumor progression represents a void in the currently available therapeutic approaches against cancer. Yet, this is the most pernicious, deadly aspect of malignancy. An alternative therapeutic approach suggested by this logic would involve the elimination of factors that would deprive local tumor cells of resources necessary for their survival. An example is presented by the concept of vascular normalization.

Another approach that initially seems counterintuitive would involve modulation of the immune response to minimize the destructive effects of an immune system attempting but incapable of completely eradicating the primary tumor. It is well known that tumors characterized by high levels of inflammation are typically associated with poorer response rates and reduced survival. This approach would likely involve re-balancing the immune response from involving mostly non-specific inflammatory processes to being more strictly antigen-specific. Such interventions may lead to stabilization of the primary tumor as an intact mass that is less likely to spawn aggressive, metastatic cells.

## Examples of Therapeutic Interventions That Fit the Paradigm of “Cancer Stabilization”

In this outline, we highlight some studies that illustrate the application of therapeutic methods, which are not strictly cytotoxic but rather target the interaction of the tumor cell with its microenvironment or adaptive mechanisms that are essential for the maintenance of a metastatic phenotype (Table 1). The list of referenced studies is far from exhaustive but attempts to capture both clinical and preclinical evidence that fits the paradigm of “cancer stabilization” for therapy.

**Table 1:** Selected therapeutic approaches that illustrate the “cancer stabilization” paradigm.

Therapeutic approach	Agents	Reference
Vascular normalization	Pro-, anti-angiogenic agents	(12, 33, 87)
	Bevacizumab	(11, 30, 73, 88, 124)

Restoration of normoxia	Hyperbaric oxygen	(1, 58, 66)
	Liposomes loaded with hemoglobin	(28)
	Microbubbles loaded with oxygen	(20)

Inhibition of epithelial-to-mesenchymal transition	Salinomycin	(98, 128)
	cAMP regulators	

Therapies targeting bioactive lipid signaling	Autotaxin inhibitors	(35, 50, 61, 97, 120)
	Fingolimod	(14, 25, 107, 108, 110)

Bone-resorption therapy	Anti-resorptive agents	(2, 45, 49, 51, 121, 129)
	Denosumab	(62, 71)

Immunotherapy	Ipilimumab	(31, 70, 76, 93, 96, 104, 125)
	Pembrolizumab/Nivolumab	(79, 109, 122, 127)
	Atezolizumab	

Metastatic-cell targeted therapy	Anti-miRNA-10b antagomirs	(5, 83, 84, 111)
		(86, 133)

### Tumor Vasculature Normalization

One approach that could limit the emergence of aggressive metastatic subclones would involve modification of the primary tumor microenvironment. Directing the primary tumor toward being a more cohesive, organized, and insulated mass through physicochemical encapsulation and/or normalization of the tumor microenvironment could prevent the escape of tumor cells or minimize the selective pressure that yields subclones capable of dissemination.

Evidence in favor of this concept comes from experience with anti-angiogenic therapy. Anti-angiogenic therapies have been investigated since the early 1970s (3–5). Traditional anti-angiogenic therapy focused on the inhibition of the formation of new vessels and the ablation of established vessels in order to limit the supply of oxygen, nutrients, and cytokines to the tumor (4, 6, 7). With specific relevance to metastasis, though, preclinical studies have shown that metastatic progression could be accelerated by anti-angiogenic therapies (8–11).

An alternative method that is sensible in the context of tumor “stabilization” relies on tumor vasculature normalization. Whereas physiological angiogenesis is important in the natural process of development, reproduction, and repair, pathological angiogenesis, seen in tumors, is characterized by the formation of abnormal vasculature that is leaky, irregular, and heterogeneous. This network of blood vessels is inefficient at effectively delivering oxygen, nutrients, and cytokines to all cancer cells within the tumor. This induces the formation of hypoxic pockets in areas to which oxygen cannot diffuse (100–200 μm away from blood vessels) (12, 13). In addition, the irregular vasculature within tumors can lead to the build-up of high interstitial fluid pressure that could drive metastatic cancer cells into blood vessels or the lymphatic fluid network (14).

Contrary to traditional anti-angiogenic approaches, the key concept of vascular normalization is to recover the balance between pro-angiogenic and anti-angiogenic stimuli and consequently induce the repair of the irregular intratumoral blood vessel network, the enhancement of intratumoral oxygenation, and the decrease of interstitial fluid pressure (15–17).

A number of preclinical and clinical trials have shown evidence of the potential success of vascular normalization therapy (18, 19). In all cases, the effects of anti-angiogenic therapies *via* vascular normalization were characterized by the increase of oxygenation, less tortuosity, thinner vessel diameter, and decreased vessel density, which resulted in less permeability, higher blood flow, lower interstitial fluid pressure, and higher perfusion. Following vascular normalization, cancer cells became more responsive to chemotherapy, radiation therapy, and immunotherapy and less prone to metastasis (18).

**Figure 1:**
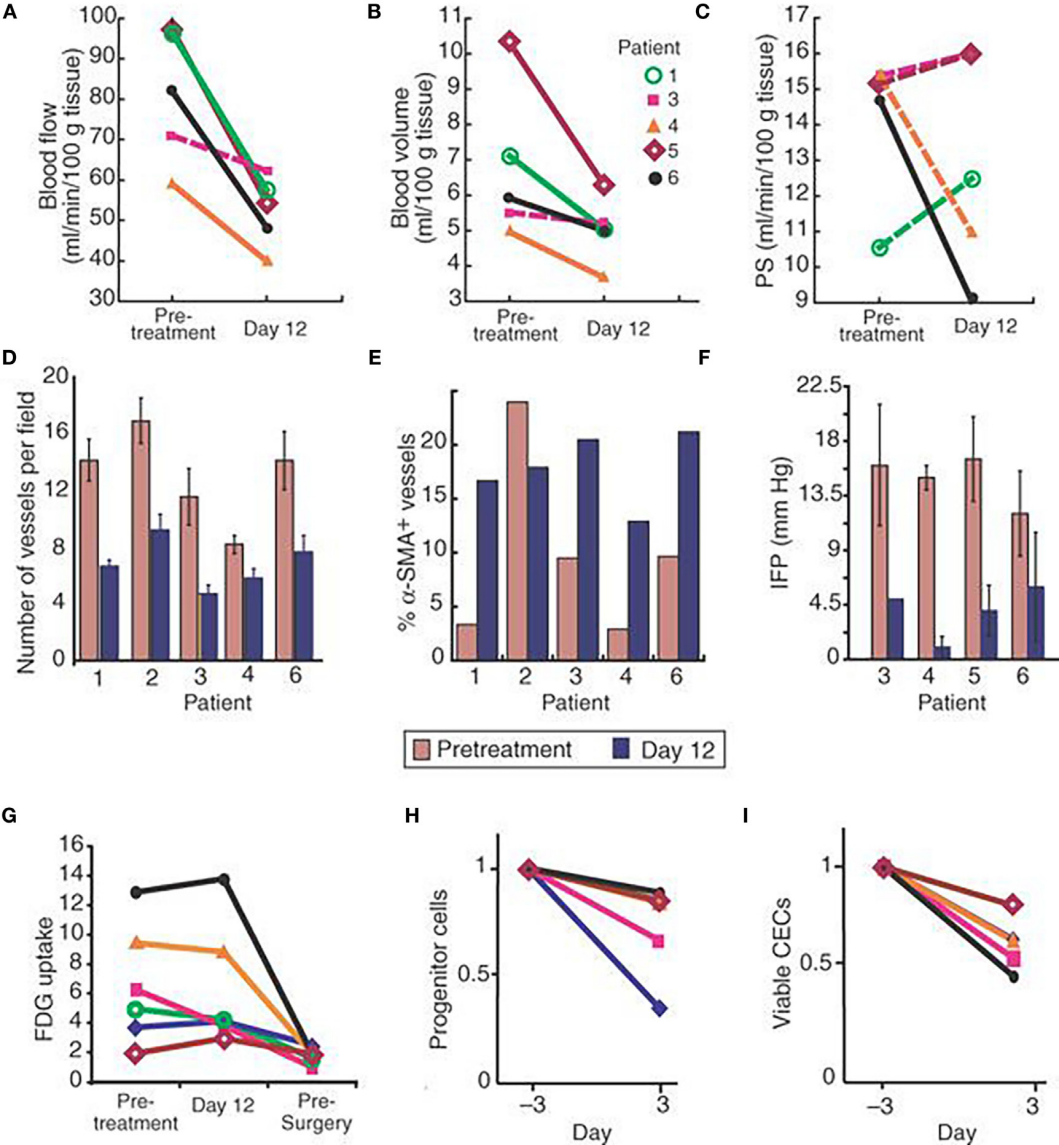
Clinical evidence that vascular normalization through a single infusion of the VEGF-specific antibody bevacizumab is effective in rectal carcinoma patients. “Parameters were obtained pretreatment and after one bevacizumab infusion. **(A−C)** Blood perfusion **(A)**, blood volume **(B)**, and permeability–surface area product [PS **(C)**]. Significant decreases after treatment are indicated by solid lines (*P* < 0.05 by *t*-test). Blood flow and blood volume decreased significantly in four of the patients. **(D)** Microvascular density. All patients showed significant decreases after treatment (*P* < 0.05 by *t*-test). **(E)** Fraction of vessels with pericyte coverage. The difference in the fraction of vessels positive for alpha-smooth muscle actin (alpha-SMA) in patient 2 was identified as an outlier by the Extreme Studentized Deviate test. Paired *t*-test analyses of the mean values that included and excluded the data of patient 2 had *P* < 0.09 and 0.001, respectively. **(F)** Mean tumor IFP decreased significantly after bevacizumab (*P* < 0.01 by paired *t*-test). **(G)** Tumor FDG uptake before treatment, on day 12 and presurgery (day 93), normalized for muscle values. On day 12 after bevacizumab treatment, a 40% decrease was observed in patient 3, and no change in the other patients. Lower levels were found in all patients before surgery except for patient 5, who had low levels throughout the treatment. In comparison to pretreatment and day 12 values, the median standard uptake value was significantly lower on day 93 (*P* < 0.01). **(H)** Circulating progenitor/stem cells (AC133+; left) and viable CECs (right) in peripheral blood. Samples were run to acquire 50,000 events in the mononuclear/lymphocyte gate. For both cell populations, bevacizumab induced a significant decrease in mean values (*P* < 0.05 by Wilcoxon signed-rank test). Key in **(B)** applies to **(A,C,G−I)**.” Reprinted from Willett et al. (29) with kind permission by Nature Publishing Group.

A key example is presented by the blockade of VEGF-related pathways for effective vascular normalization through the normalization of the endothelial layer (20–23). In clinical trials, chemotherapy combined with bevacizumab (humanized monoclonal antibody against VEGF) improved survival in patients with metastatic cancer of the breast, kidneys, colon, and lung (24–28). This seemingly unexpected effect was explained by a hypothesis first proposed by Rakesh Jain from Harvard Medical School. In their seminal studies, the authors observed time- and dose-dependent transient vascular normalization following treatment with the anti-VEGF agent in rectal carcinoma patients. A single infusion of bevacizumab mediated a decrease in vascular volume, tumor perfusion, and microvascular density, resulting in an overall reduction in interstitial fluid pressure (Figure 1). These data led to the conclusion that VEGF inhibition mediates an antivascular effect in clinical cancer (29).

In addition to the patho-physiological changes induced by vascular normalization, there is also enhancement of drug delivery. The Enhanced Permeation-Retention (EPR) effect is very effective for the delivery of large-sized particles or protein conjugates *via* passive targeting. The leaky vasculature, the main route of drug delivery based on the EPR effect, is repaired during the process of vascular normalization, leading to improvement in the delivery of large particles to tumors, as shown in models of breast cancer (30).

The recent past has revealed another nuance in the utility of vasculature normalization for cancer therapy. Specifically, convincing evidence is emerging of the synergism between immunotherapy and vascular normalization therapy. On the one hand, vascular normalization minimizes hemodynamic obstacles to T lymphocyte infiltration. On the other hand, CD4^+^ T lymphocyte inactivation leads to increased vessel tortuosity, suggesting reciprocal regulation between T_H_1 cells and vascular status, as shown in models of mammary carcinoma (31).

Despite its promise, vascular normalization has certain limitations. First, it is not a permanent or long-lasting condition, but a transient state. Pharmacologically, it has a “time window” that is established 1–2 days from the time point of anti-angiogenic treatment. After this “time-window,” vascular normalization ends irreversibly and does not recover (32). Second, vascular normalization can be established only when pro- and anti-angiogenic factors are well balanced, which requires precise control over the dose of anti-angiogenic therapeutics.

### Restoration of Normoxia

An alternative approach to tumor vascular normalization would involve restoration of normoxia in the tumor microenvironment. When the concentration of oxygen in the tumor is below normal (normoxia), the tumor is defined as “hypoxic” (33, 34, 35). Hypoxia stimulates cancer cells to secrete signaling molecules for the activation of hypoxia-associated pathways, mainly including HIF, PI3K/AKT/mTOR, MAPK (or ERK), and NFkB (36–40).

The HIF pathway is most important in cancer cell proliferation, survival, apoptosis, metabolism, migration, and inflammation. In normoxia, oxygen sensors (PHD and FIH-1) regulate the expression level of HIF-α subunits (HIF-1 α, HIF-2 α, and HIF-3 α) in endothelial cells to convert hydroxylated HIF- α subunits, which are consequently degraded by proteasomes. In contrast, oxygen sensors are deactivated in hypoxia and cannot hydroxylate HIF- α subunits. Unhydroxylated HIF- α subunits are stable. They move to the nucleus, form dimers with HIF-β, and initiate transcription of compensatory targets (41–44). The HIF pathway regulates hundreds of genes and facilitates tumor growth by the promotion of metabolism and angiogenesis (45, 46, 47, 48, 49). In addition, HIF-α promotes metastasis by regulating epithelial-to-mesenchymal transition (EMT) through ZEB-1, ZEB-2, E-cadherin, and TCF3, and migration/invasion through MMP-2 and MMP-9, CXCR4, CAIX, and LOX (50–54). Also, HIF-α interacts with other oncologic pathways. For example, the combination of the HIF-1α and NFkB pathways is associated with the regulation of more than 1,000 oncologic or inflammatory genes. Furthermore, cancer cells that experience EMT transition in hypoxia are more resistant to chemotherapy as well as to radiation therapy. In normoxia, oxygen mediates sensitivity to radiotherapy by reacting with free radicals produced by ionized radiation. This mechanism triggers DNA damage. In contrast, cancer cells in hypoxic conditions are less sensitive to radiation due to limited generation of DNA radicals (55).

One approach that attempts to restore normoxia is hyperbaric oxygen (HBO) therapy. The goal of HBO therapy is to improve or cure disorders by increasing oxygen levels in plasma and tissue (56). HBO therapy showed a significant increase in pO_2_ in tumor tissue, which was preserved clinically for 30 min (57–60). Consequently, HBO therapy induced the activation of the pro-apoptotic MAPK pathway and the downregulation of members of the anti-apoptotic ERK pathway in hematopoietic cells. The same results were obtained in preclinical studies demonstrating the induction of cell death and the inhibition of cell proliferation (61–63). With regard to metastasis, HBO therapy has resulted in the induction of mesenchymal-to-epithelial transition (MET), associated with a less invasive tumor cell phenotype. This result implies that HBO therapy could be useful for the potential inhibition of the metastatic process (64, 65).

A more targeted therapeutic approach involves oxygen delivery to tumors using delivery vehicles, including microbubbles and hemoglobin encapsulated liposomes (66–68). The use of delivery vehicles could reduce the risk of oxygen toxicity or eliminate undesirable off-target effects that could be associated with HBO therapy (69, 70).

Microbubbles are typically utilized as contrast agents for ultrasound imaging and sonodynamic therapy. They comprise a lipid, protein, natural, or synthetic polymer as an outer shell and a gaseous core (71–76). The microbubble allows free gas diffusion across its shell, allowing one to regulate the amount of oxygen delivered to tissue depending on how much dissolved gas is found in the local microenvironment. Microbubbles, therefore, acquire oxygen in the lungs and release it in hypoxic tissues. In preclinical studies, oxygen microbubbles reduced the expression of HIF-1α by 50% in cancer cells cultured in hypoxic conditions. This resulted in significant reduction in tumor volume after sonodynamic therapy (73, 77).

**Figure 2:**
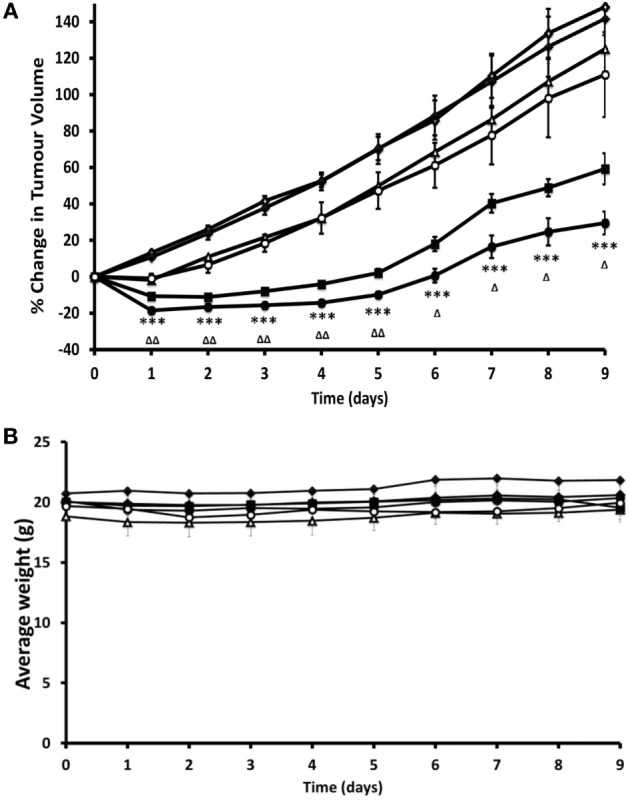
Preclinical evidence that restoration of normoxia through treatment with oxygen-loaded microbubbles (O_2_MB-RB) is effective in a murine model of pancreatic cancer. Ectopic human xenograft BxPC-3 tumors were established in the rear dorsum of SCID mice. Plot of **(A)** % change in tumor volume and **(B)** average body weight for mice treated with (i) no treatment (open diamonds), (ii) ultrasound only (filled diamonds), (iii) gemcitabine (open triangles), (iv) O_2_MB-RB/O_2_MB-5FU mix − US (open circles), (v) O_2_MB-RB + US (filled squares), (vi) O_2_MB-RB/O_2_MB-5FU mix + US (filled circles). Not shown for ease of illustration are treatments with 5-FU alone, O_2_MB-RB − US, O_2_MB-5FU + US, O_2_MB-5FU − US. The RB, 5-FU and gemcitabine concentrations were kept constant in each case at 0.184 mg/kg (90.8 µM), 0.115 mg/kg (440 µM), and 0.264 mg/kg (440 µM), respectively. Ultrasound treatment was delivered for 30 s at frequency of 1 MHz, an ultrasound power density of 3.0 Wcm^−2^ and a duty cycle of 50%, pulse frequency = 100 Hz. Error bars represent ± SE where *n* = 4. *P < 0.05, **P < 0.01, and ***P < 0.001 for (vi) compared to (i) and ^Δ^*P* < 0.05, ^ΔΔ^*P* < 0.01, and ^ΔΔΔ^*P* < 0.001 for (vi) compared to (v). “These results reveal a dramatic reduction in tumor volume for mice treated with the combined SDT/antimetabolite therapy compared to either gemcitabine or 5-FU treatment alone.” Reprinted from Mcewan et al. (78) with kind permission by Elsevier Publishing Group.

We would like to specifically highlight a more recent preclinical study, which described the design and application of oxygen-loaded microbubbles (O2MB) for pancreatic cancer therapy. O2MBs were designed to incorporate either Rose Bengal or 5-fluorouracil. Treatment with either type of micro-bubble in human xenograft models of pancreatic cancer resulted in reduction in tumor growth, illustrating the capability of microbubble-delivered oxygen to the tumor to enhance therapeutic efficacy, albeit in a somewhat artificial setting of a subcutaneously implanted model (Figure 2).

In addition to the methods described above, other approaches for targeting the hypoxic phenotype have been described. These include targeting of HIFs or other pathways important in hypoxia. However, these approaches rely on inducing cell death in a hypoxic environment or involve direct or indirect HIF inhibition. These studies are discussed in detail in Ref. (79).

Combined, these results support the hypothesis that restoring normoxia in tumor tissue can represent a component in a therapeutic approach aimed at normalizing the tumor microenvironment for the suppression of metastasis.

### Inhibiting Epithelial to Mesenchymal Transition

During EMT, epithelial cells assume a mesenchymal phenotype and are capable of disengaging from the basement membrane, migrating and invading surrounding tissue (80). In addition, cells that undergo EMT often acquire stem cell-like characteristics including tumor cell initiating properties and chemoresistance (81). It is not surprising then that tumors with EMT features have been associated with poor prognosis in several cancers including breast cancer (82), pancreatic adenocarcinoma (83), hepatocellular carcinoma (84), gastric cancer (85), and non-small cell lung cancer (86). Over the last decade, evidence has shown that EMT can be initiated by miRNAs as well as several transcription factors, leading to the activation of several downstream pathways (87).

Targeting the EMT pathway has thus emerged as an area of great therapeutic interest. EMT-targeted therapies could be used to inhibit metastasis in high-risk patients or to partially reverse existing metastatic disease (88). Unfortunately, several challenges exist including difficulties in targeting the miRNAs and transcription factors that induce EMT and an apparent redundancy in the pathways activated by EMT. However, a few approaches have emerged in the last few years.

**Figure 3:**
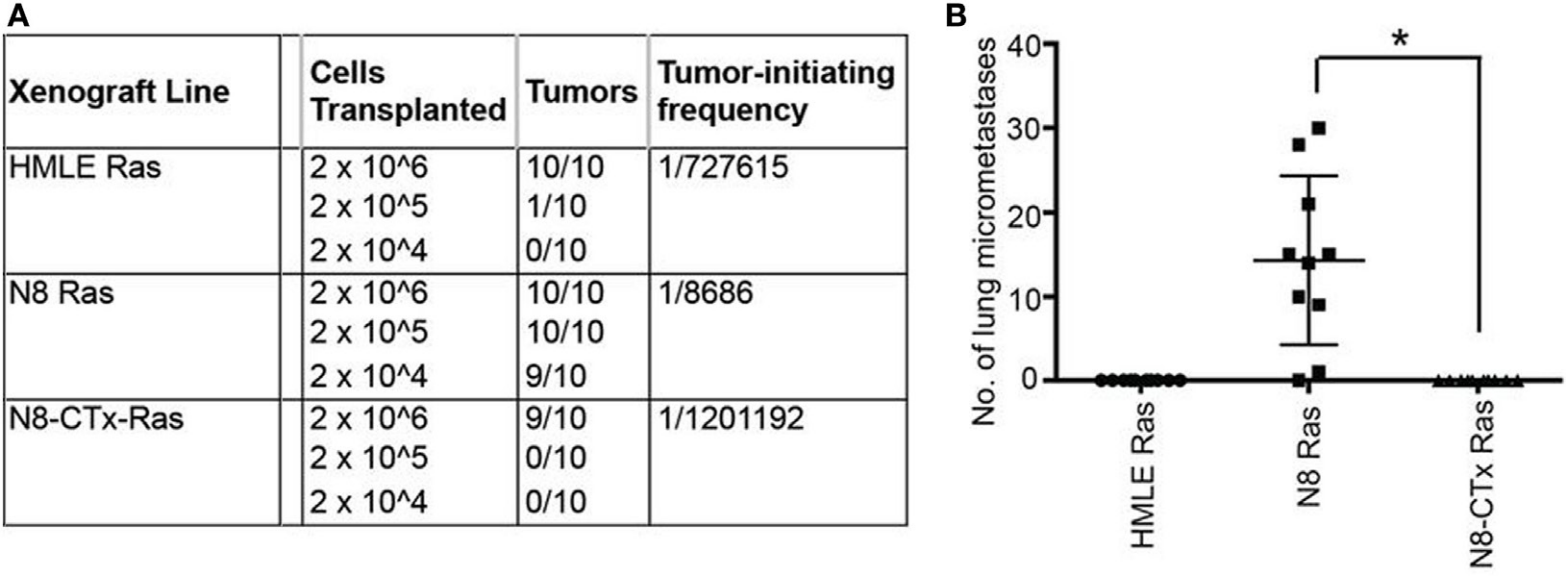
Preclinical evidence that increases in intracellular levels of the second messenger, cAMP, lead to activation of protein kinase A and cause mesenchymal human mammary epithelial cells to revert to their epithelial state, resulting in inhibition of tumor-initiating and metastatic potential. The authors “transplanted at limiting dilutions HMLE-Ras immortalized human mammary epithelial cells and their spontaneously arising mesenchymal derivatives, termed NAMEC8 (N8-Ras) cells, as well as N8-CTx-Ras cells that were mesenchymal-to-epithelial transition (MET) reverted by treatment with cholera toxin (CTx). Cells were implanted into the mammary fat pads of NOD/SCID mice. **(A)** As anticipated, the frequency of tumor-initiating cells in the N8-Ras cells was far greater than in the HMLE-Ras cell population, in this case, 100-fold higher. Significantly, the N8-CTx-Ras cells were as inefficient at tumor-initiation as the HMLE-Ras cells. **(B)** The primary tumors that arose upon orthotopic mammary stromal fat pad implantation of N8-Ras tumors spawned 20–30 micrometastases in the lungs by 12 weeks following implantation. This property was completely lost upon induction of an MET by CTx treatment prior to transplantation.” Reprinted from Pattabiraman et al. (90) with kind permission by the American Association for the Advancement of Science.

For example, salinomycin was identified in a screen to detect drugs that would inhibit EMT-induced cancer stem cells, and subsequent studies showed that it inhibits breast cancer metastasis as well (89). More recently, Pattabiraman et al. proposed a “differentiation therapy” whereby increases in intracellular levels of the second messenger, cAMP, led to activation of protein kinase A (PKA) causing mesenchymal human mammary epithelial cells to revert to their epithelial state through a MET (90). Induction of MET caused a dramatic loss not only in their ability to metastasize but also in their tumor-initiating properties (Figure 3). Thus, salinomcyin and “dedifferentiation therapies” could be used to reduce metastasis.

### Therapies Targeting Bioactive Lipid Signaling

Several small bioactive lipids may also be important mediators of metastasis. Lysophosphatidic acid (LPA) is produced by the soluble enzyme autotaxin (ATX) and can signal through a family of high affinity G protein-coupled LPA receptors that includes six members (LPAR1-6). Signaling through these receptors can induce EMT (91), and overexpression of ATX and LPAR1-6 are common in many cancer types and associated with increased metastases in transgenic animals (92). LPA can be dephosphorylated by lipid phosphate phosphatases (LPPs), which leads to its degradation. LPP1 expression is typically decreased in tumors and restoring its expression leads to decreased metastasis in mice (93).

Autotaxin inhibitors have been shown to reduce lung metastases after systemic injection of the mouse B16F10 melanoma cell line (94). Interestingly, reduced lung metastases were also observed in LPAR1 knockout mice (95). ATX/LPA signaling is an important driver of pulmonary (96), renal (97), and liver fibrosis (98). An ATX inhibitor and an LPAR1 antagonist are currently in trials for idiopathic pulmonary fibrosis (NCT02738801 and NCT01766817) and could be potentially repurposed as an anti-metastases therapy.

Another bioactive lipid that has been associated with metastasis is sphingosine-1-phosphate (S1P). Sphingosine is released from ceramide through the actions of ceramidase and then is phosphorylated by sphingosine kinase to generate S1P. S1P can be dephosphorylated back to sphingosine by sphingosine phosphatases, degraded by sphingosine phosphate lyase, or exported out of the cell by a transporter, called spinster 2 (SPNS2), where it can signal to cells through 5 sphingosine receptors (S1P1-5).

Lymphocytes sense S1P gradients in the plasma through S1P1 to egress from secondary lymphoid tissues. FTY720 (Fingolimod) was developed as an immunomodulating drug mostly for treating the relapsing form of multiple sclerosis. Fingolimod is phosphorylated forming fingolimod-phosphate. In its phosphorylated form, it can activate lymphocyte S1P1 and ultimately induce S1P1 downregulation, sequestering lymphocytes in lymph nodes and preventing autoimmunity. Interestingly, in animal models, FTY720 has been shown to reduce metastases of several cancers including breast cancer (99, 100), liver cancer (101, 102), and cholangiocarcinoma (103).

**Figure 4:**
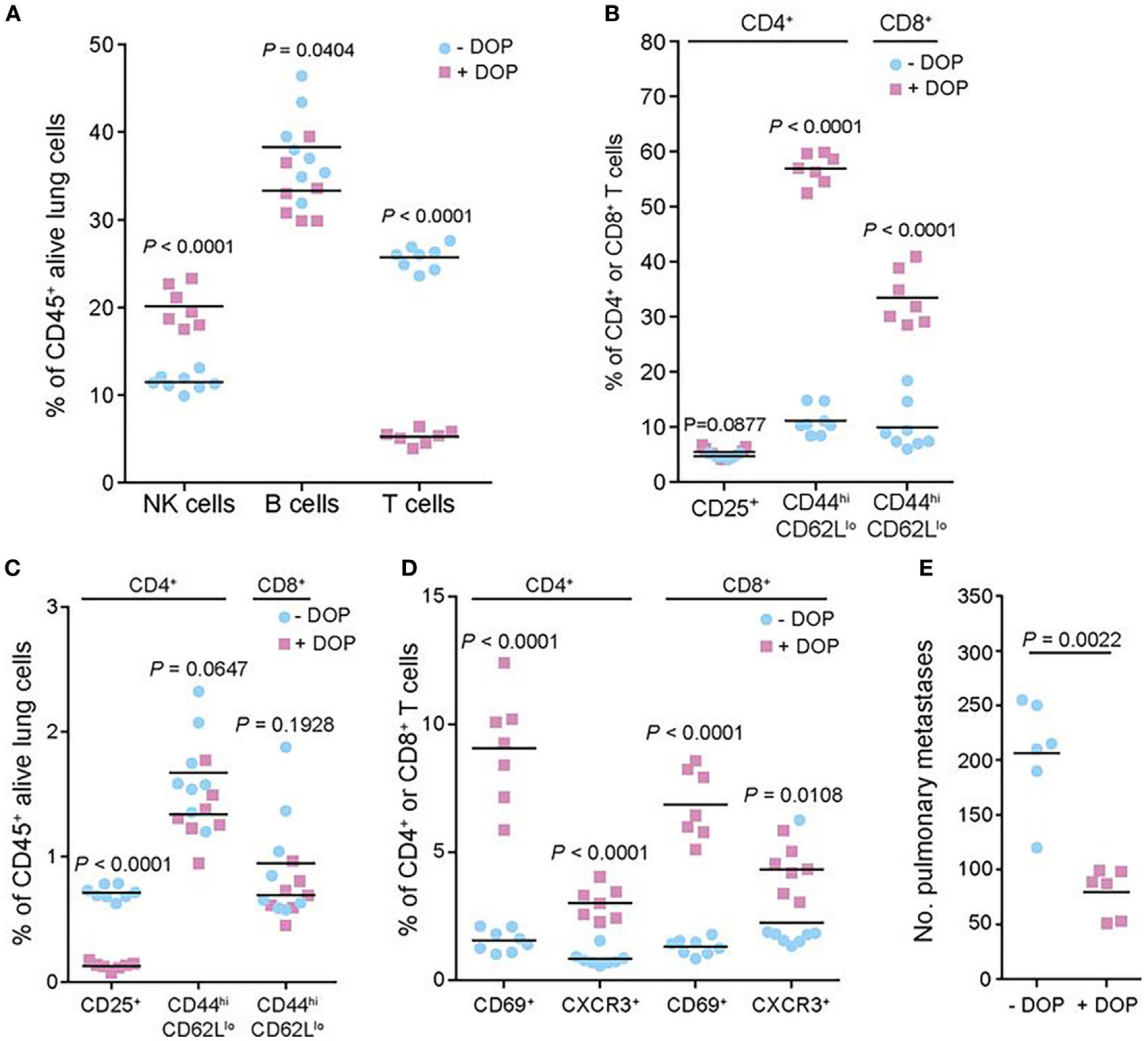
Preclinical evidence in a murine model of melanoma that lipid signaling is an important player in metastasis, which could be potentially targeted for therapy. The authors performed a genome-wide *in vivo* screen of 810 mutant mouse lines, which resulted in the identification of host genes that regulate metastatic colonization in the lung after injection of B16 melanoma cells. Their screen identified 23 hits including the sphingosine-1-phosphate (S1P) transporter Spns2. In the studies shown here, the authors manipulated the S1P axis pharmacologically by inhibiting S1P lyase, which degrades S1P, using 4′-deoxypyridoxine (DOP), a compound previously shown to increase lymphoid tissue S1P levels and induce a circulating lymphopenia. The studies shown here demonstrated that deletion of Spns2 by DOP treatment created a circulating lymphopenia with increased numbers of natural killer cells and effector T cells in the lung, which effectively prevented lung colonization. **(A,C)** Number of leukocytes and T cell subsets present in the lungs of B16-F10-dosed glucose- or DOP-treated wild-type male mice presented as the percentages of viable CD45^+^ lung leukocytes. **(B,D)** Number of leukocytes and T cell subsets presented as the percentages of viable parent CD4^+^ or CD8^+^ T cells. **(E)** Experimental metastasis assay in B16-F10 dosed glucose- or DOP-treated wild-type female mice. Reprinted from van der Weyden et al. (104) with kind permission by the Nature Publishing Group.

More recently, van der Weyden et al. performed a genome-wide *in vivo* screen of 810 mutant mouse lines, which resulted in the identification of host genes that regulate metastatic colonization in the lung after injection of B16 melanoma cells (104). Their screen identified 23 hits including the S1P transporter Spns2. Further studies demonstrated that deletion of Spns2 created a circulating lymphopenia with increased numbers of natural killer (NK) cells and effector T cells in the lung, which effectively prevent lung colonization (Figure 4). Thus, lipid signaling seems to be an important player in cancer metastasis as well, which could be potentially targeted through several approaches.

### Bone-Resorption Therapy

In addition to modifying the primary tumor microenvironment to prevent the emergence and escape of invasive metastatic cells, it is possible to modify the local tissue microenvironment of a distant vital organ, such as the lungs, liver, brain, or bone. Modification of the distant organ microenvironment would make that organ inhospitable to the metastatic tumor cell and limit the establishment and growth of metastatic lesions.

One organ that is commonly colonized in the process of metastasis is bone. Bone provides a unique environment that is characterized by dynamic interplay between multiple cell types including mesenchymal cells (osteocytes, osteoblasts, and adipocytes), hematopoietic cells (immune cells and osteoclasts), endothelial cells, and pericytes (105). In the process of metastasis, circulating tumor cells attach to bone and initiate a cascade of signaling events that promote tumor cell survival in this new microenvironment.

Tumor cells can stimulate the osteoclast lineage to accelerate differentiation, which results in more rapid osteoclastic bone resorption than osteoblastic bone formation. This excessive bone degradation forms cavities in bone where tumor cells can settle to form osteolytic metastatic lesions. Conversely, tumor cells can also excrete cytokines to stimulate osteoblast differentiation and deposition of new bone tissue, causing faster bone formation than resorption, and resulting in excessive bone growth at the sites occupied by metastatic lesions (106).

Therapeutic approaches include anti-resorptive drugs, which have shown excellent therapeutic efficacy in malignancies, such as prostate, breast, lung, and multiple myeloma (107–112). A specific example is presented by bisphosphonates, which are ingested by osteoclasts and result in osteoclast cytotoxicity, effectively limiting osteoclastic bone resorption, as shown in mammary cancer models (113). Interestingly, antibodies developed for the treatment of osteoporosis inhibited bone metastasis by remodeling bone structure in breast cancer patients (114).

**Figure 5:**
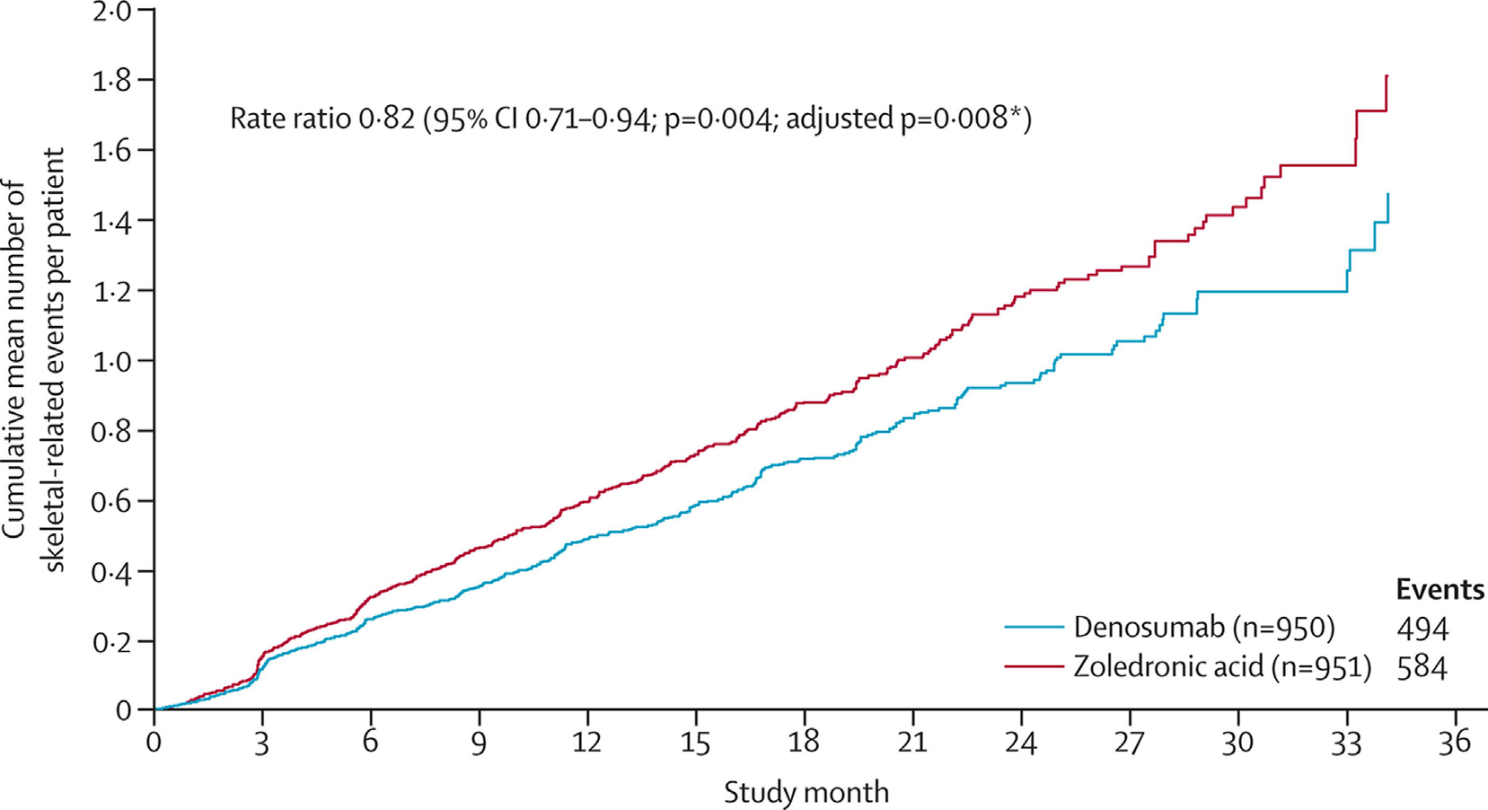
Clinical evidence that anti-osteoclastic therapy can be effective in patients with castration-resistant prostate cancer. “Time to first and subsequent on-study skeletal-related events in prostate cancer patients treated with denosumab vs. zoledronic acid.” Reprinted from Fizazi et al. (115) with kind permission by Elsevier.

A related therapeutic agent targets a molecule, called RANKL, which is secreted by osteoblasts. RANKL attaches to RANK (located on osteoclasts) and stimulates osteoclastic activity. This therapeutic agent (Denosumab) is a human monoclonal antibody against RANKL, which prevents the interaction between RANKL and RANK, and inhibits osteoclastic activity. Denosumab is FDA approved and has shown promising results in patients, including a prolonged time to skeletal-related event (115). In a phase 3 study, denosumab was compared to the bisphosphonate zoledronic acid for the treatment of castration-resistant prostate cancer. The median time to “first on-study skeletal-related event” was 20.7 months with denosumab vs. 17.1 months with zoledronic acid (Figure 5), leading the authors to conclude that denosumab represents a viable treatment option against bone metastases from castration-resistant prostate cancer.

Additional therapeutic approaches for bone metastatic disease include radioisotopes, which incorporate into bone and deliver ionizing radiation, chemotherapy, hormonal therapy, and more modern targeted immunotherapy. However, these approaches primarily target the metastatic tumor cell and are beyond the scope of the current review. For a thorough review on this topic, we direct the reader to Gdowski et al. (116).

### Immunotherapy

The last decade has witnessed impressive progress in the field of immunotherapy. Recent evidence of robust clinical responses has been seen in multiple malignancies, including metastatic melanoma, non-small cell lung cancer, head and neck cancer, renal cell carcinoma (RCCs), breast cancer, and hematological malignancies (117).

Partly, the reason behind this success is the capacity of these interventions to suppress tolerance to tumor antigens. The immunotherapeutic approaches available now include cancer vaccines, checkpoint inhibitors, oncolytic viruses, and adoptive T-cell or NK cell transfer. Here, we will focus on immune checkpoint inhibitors because they fit more closely the paradigm of restoring immunological homeostasis in the tumor microenvironment.

It is well-known that tumor cells have developed efficient mechanisms to escape immune recognition. These include tolerance induction, immune evasion, and interference with T cell signaling. To avoid immune recognition, tumor cells highjack the body’s system of checks and balances that controls T cell mediated cellular immunity.

Specifically, when the T cell receptor (TCR) of a T cell recognizes foreign antigens in the context of the major histocompatibility complex, additional binding events modulate the ensuing response through co-stimulatory factors, such as CD28 (serving to amplify the signal by binding to CD80/CD86 on antigen-presenting cells) or immune checkpoint molecules, such as CTLA-4 or PD-1 (to suppress the signal by binding to CD80/CD86 on antigen-presenting cells or PD-L1 on tumor cells or activated macrophages). Upregulation of PD-L1 on tumor cells leads to engagement of PD-1 on T lymphocytes and suppression of the cytotoxic immune response even in the presence of proper recognition of tumor antigen by the TCR. Conversely, pharmacological PD-1/PD-L1 inhibition prevents the PD-1/PD-L1 interaction, facilitating the mounting of an effective cytotoxic response.

A thorough review on this subject can be found in Alsaab et al. (117) and Farkona et al. (118). Here, we would like to highlight the clinical progress that has been made using CTLA-4 and PD-1 inhibitors. Ipilimumab, an anti-CTLA-4 antibody, was approved by the FDA in 2011, following successful phase III clinical trials in patients with metastatic melanoma (119–121). More recently, testing in clinical trials was initiated for the treatment of non-small cell lung carcinoma (NSCLC), small cell lung cancer, bladder cancer, and metastatic hormone-refractory prostate cancer. Despite this early success, it has become clear that CTLA-4 blockade is likely to help only a small fraction of patients and could result in grades 3–5 (severe) immune-related adverse events in 10–35% of patients (122). In addition, response to ipilimumab may take several months to manifest, making it difficult to assess therapeutic efficacy (118, 123).

**Figure 6:**
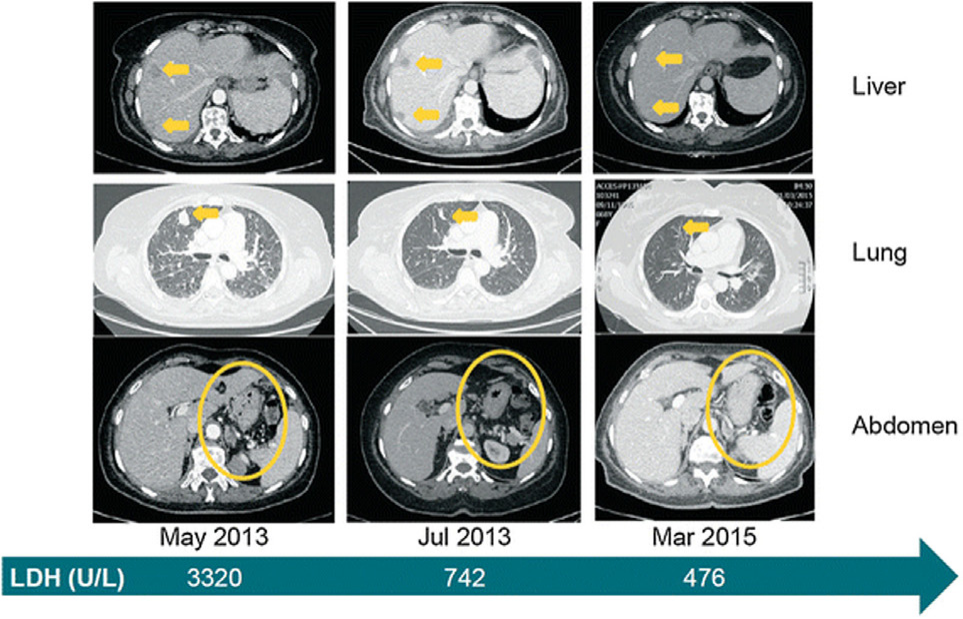
Clinical evidence that treatment with the immune checkpoint inhibitor, nivolumab is effective against metastatic mucosal melanoma. Systemic clinical response to nivolumab treatment. Computed tomography images from baseline (May 2013), 2 months into treatment and approximately 2 years later. *Yellow arrows or circles* indicate metastatic foci and their complete disappearance by March 2015. The *green arrow* depicts the level of LDH at the selected time points. *LDH* lactate dehydrogenase. Reprinted from Ascierto et al. (126) with kind permission by Springer Berlin Heidelberg.

Promising clinical results have been obtained using antibodies against the PD1–PD-L1 axis. These include pembrolizumab (previously named as lambrolizumab; anti-PD1) and nivolumab (anti-PD1) (124). A lot of the excitement over therapy with checkpoint inhibitors rests on the robust therapeutic responses seen in some patients with these agents. In clinical trials for melanoma, nivolumab was associated with often durable clinical responses (125). A case report is presented in Figure 6. The study illustrates successful therapy with nivolumab in a patient with metastatic mucosal melanoma who ultimately achieved a durable complete response (126).

The anti-PD-L1 antibody atezolizumab has shown promise in a wide range of malignancies, including colon, lung, renal cell, gastric, head and neck cancer, and melanoma. Clinical approval has been obtained for pembrolizumab and nivolumab for melanoma and NSCLC, whereas nivolumab has also been approved for RCC (121) and metastatic urothelial carcinoma [reviewed in Farkona et al. (118)].

In addition to the robust durable therapeutic responses that have been achieved with PD-1-PD-L1 inhibitors, these agents have also displayed a manageable toxicity profile. Unlike CTLA-4 inhibitors, PD-1 inhibitors are associated with mild immunostimulation that can be treated with supportive care and steroid administration (118).

Taken together, these observations suggest that metastatic cancer could be managed successfully by restoring homeostasis in the immunological microenvironment of the tumor cell. The unprecedented success of cancer immunotherapy hints at the potential of therapeutic approaches that aim to “correct” or “normalize” microenvironmental aspects of cancer emergence and progression, as opposed to interventions that are purely cytotoxic to the tumor cell.

### Metastatic-Cell Targeted Therapy

A successful approach for treating metastasis would invariably include intervention at the level of the metastatic cell. One unique property of metastatic tumor cells that could be targeted for therapy is their resistance to anoikis. Anoikis is a type of programmed cell death, which occurs when anchorage-dependent cells detach from the ECM. Healthy cells usually stay associated with the tissue to which they belong since their survival depends critically on communication between proximal cells and the ECM. When these cells are detached from the ECM, they invariably undergo anoikis. By contrast, metastatic tumor cells can become resistant to anoikis. This capability is key to their escape from the primary tumor, survival in the lymphatics or circulation, and ultimately, colonization of other vital organs (12, 127–129). Based on this knowledge, it becomes clear that by restoring sensitivity to anoikis in metastatic tumor cells, one could trigger programmed cell death in these cells in a way that is efficient and spares healthy cells.

In our search for a therapeutic target that fits this profile, we focused on microRNA-10b as a key initiator of metastasis and driver of resistance to anoikis. In a seminal study, Ma et al. showed that microRNA-10b (miR-10b) was overexpressed in metastatic breast cancer cells and could initiate invasion and metastasis in otherwise non-metastatic cells (130). In patients, miR-10b has been implicated in metastasis and disease outcome of multiple malignancies including lung, breast, gastric, colorectal, bladder, ovarian, pancreatic, hepatocellular, and brain cancer, to name a few (130–132).

In our own studies, we identified miRNA-10b as a master regulator of the viability of metastatic tumor cells. We determined that miR-10b not only promotes the capacity of tumor cells to migrate and invade surrounding tissue (become metastatic) but also serves as a powerful master regulator of the viability of these cells (133–135). Detailed mechanistic studies confirmed the existence of a miR-10b-triggered pathway that regulates the viability and proliferation of tumor cells only after they have acquired the ability metastasize, pointing to miR-10b as a driver of resistance to anoikis (134).

**Figure 7:**
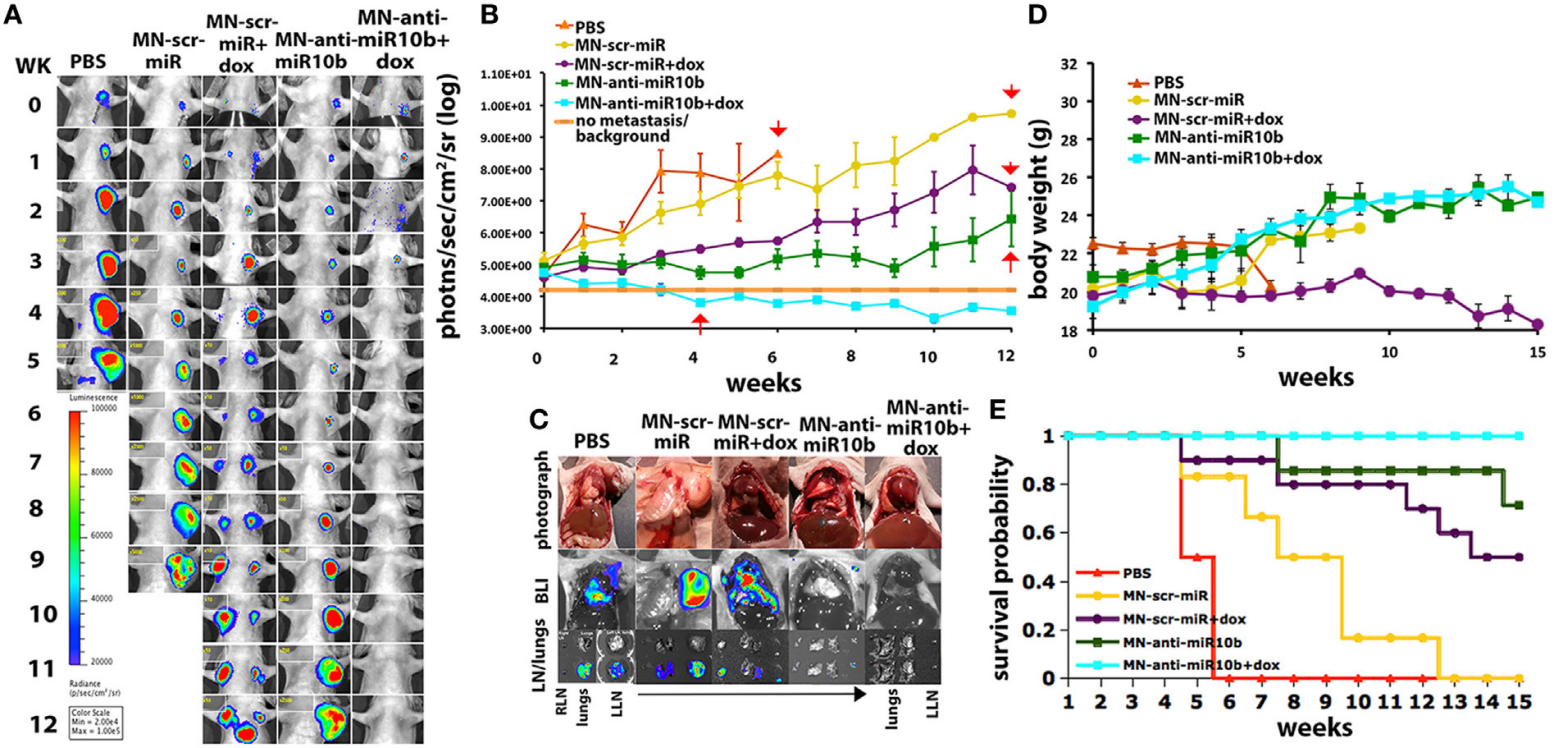
Preclinical evidence that metastatic-cell targeted therapy is effective in a murine model of metastatic breast cancer. “Metastatic burden and survival of mice orthotopically implanted with human breast cancer cells and treated with the miR-10b inhibitor MN-anti-miR-10b and low-dose doxorubicin (Dox). **(A)** Representative bioluminescence images of metastatic burden showing complete regression of metastases in animals treated with MN-anti-miR-10b and doxorubicin. **(B)** Quantitative analysis of metastatic burden from all treatment groups, indicating complete regression of metastatic burden in the lymph nodes of experimental animals treated with MN-anti-miR-10b and doxorubicin after just four weekly treatments. Background counts are derived from non-tumor-bearing animals. **(C)** *Ex vivo* BLI showing the absence of detectable lymph node or lung metastases in mice treated with MN-anti-miR-10b and doxorubicin. In animals treated with MN-anti-miR-10b alone, there were lymph nodes but not lung metastases. In all other groups, there were both lymph node and lung metastases. **(D)** Animal weight. The groups treated with MN-anti-miR-10b with or without doxorubicin continued to gain weight throughout the time course of the study. **(E)** Mortality. Only in the group of animals treated with MN-anti-miR-10b and doxorubicin, there was no mortality from carcinoma. Data, average ± SEM; within-subjects ANOVA: *P* < 0.05. PBS, *n* = 2; MN-scr-miR, *n* = 6; MN-scr-miR + Dox, *n* = 10; MN-anti-miR-10b, *n* = 7; MN-anti-miR-10b + Dox, *n* = 10.” Reprinted from Yoo et al. (134) with kind permission by AACR.

This knowledge allowed us to develop a therapeutic strategy based on miR-10b inhibition. The specific inhibition of miR-10b was achieved using inhibitory oligonucleotides (LNA-based antagomirs) delivered to metastatic sites by dextran-coated iron oxide nanoparticles (termed MN-anti-miR10b). We demonstrated that MN-anti-miR10b could completely prevent the formation of *de novo* metastases (133) and, when combined with low-dose chemotherapy, caused complete and persistent regression of local lymph node metastasis in a murine breast cancer model (Figure 7) (134).

In a model of Stage IV metastatic breast cancer, we found that a weekly treatment protocol with MN-anti-miR-10b and low-dose doxorubicin demonstrated complete regression of pre-existing lung metastases in 65% of the animals and inhibition of multiple organ metastases in 94% of the animals. This translated into a significant reduction in cancer mortality in animals treated with MN-anti-miR10b and low-dose doxorubicin relative to control groups, including a group treated with monotherapy of standard dose doxorubicin, used to model standard-of-care (135).

These studies illustrate the potential of therapeutic approaches that target the unique capability of metastatic tumor cells to survive in the absence of “healthy” cell–cell and cell–stroma interactions and outside of their natural microenvironment. Combined with efficient delivery vehicles, similar targeted therapeutic approaches could provide a means to target cancer on a systemic level.

## Conclusion

The recent past has seen impressive progress in the field of cancer therapy. Still, the outcomes for people diagnosed with advanced metastatic cancer are poor. There has been minimal progress in the overall survival of stage IV malignancy and 5-year survival rates are still below 20% for cancers such as pancreatic, bile duct, colorectal, NSCL, liver, gastric, ovarian, and esophageal cancer. These poor outcomes highlight the need to develop strategies different from the traditional cytotoxic approach to cancer therapy that has dominated the field over the past century.

This review outlines a new methodology that consists of three major components. The first component involves modification of the primary tumor microenvironment in order to limit the emergence and escape of invasive tumor cells. The second component entails modification of the microenvironment of the distant metastatic organ in order to make it less hospitable to metastatic tumor cells. The third component includes disruption of anchorage independence and immune tolerance to the tumor cells with the goal of eliciting systemic regression of metastatic burden. These interventions, along with surgical resection, could conceivably lead to robust therapeutic outcomes in patients with otherwise poor prognosis. As outlined here, first steps in this direction have already been made. Still, the general concept of “stabilization” rather than “eradication” of cancer, as a companion to surgical resection, can spur additional research that could have a transformative impact on the management of metastatic disease.

## Author Contributions

BY, BF, and ZM performed the conceptualization, the literature search, the data extraction, and composed the manuscript. All the authors contributed substantially to the final manuscript and approved the final version.

## Conflict of Interest Statement

ZM is a Founder, Director, and Scientific Advisory Board Member of TransCode Therapeutics, Inc. The other authors declare that the research was conducted in the absence of any commercial or financial relationships that could be construed as a potential conflict of interest.
